# Genome-wide analysis of alternative splicing of pre-mRNA under salt stress in Arabidopsis

**DOI:** 10.1186/1471-2164-15-431

**Published:** 2014-06-04

**Authors:** Feng Ding, Peng Cui, Zhenyu Wang, Shoudong Zhang, Shahjahan Ali, Liming Xiong

**Affiliations:** Biological and Environmental Sciences and Engineering Division, King Abdullah University of Science and Technology (KAUST), Thuwal, 23955-6900 Saudi Arabia

**Keywords:** Alternative splicing, Pre-mRNA, SR proteins, Salt stress, *Arabidopsis thaliana*

## Abstract

**Background:**

Alternative splicing (AS) of precursor mRNA (pre-mRNA) is an important gene regulation process that potentially regulates many physiological processes in plants, including the response to abiotic stresses such as salt stress.

**Results:**

To analyze global changes in AS under salt stress, we obtained high-coverage (~200 times) RNA sequencing data from *Arabidopsis thaliana* seedlings that were treated with different concentrations of NaCl. We detected that ~49% of all intron-containing genes were alternatively spliced under salt stress, 10% of which experienced significant differential alternative splicing (DAS). Furthermore, AS increased significantly under salt stress compared with under unstressed conditions. We demonstrated that most DAS genes were not differentially regulated by salt stress, suggesting that AS may represent an independent layer of gene regulation in response to stress. Our analysis of functional categories suggested that DAS genes were associated with specific functional pathways, such as the pathways for the responses to stresses and RNA splicing. We revealed that serine/arginine-rich (SR) splicing factors were frequently and specifically regulated in AS under salt stresses, suggesting a complex loop in AS regulation for stress adaptation. We also showed that alternative splicing site selection (SS) occurred most frequently at 4 nucleotides upstream or downstream of the dominant sites and that exon skipping tended to link with alternative SS.

**Conclusions:**

Our study provided a comprehensive view of AS under salt stress and revealed novel insights into the potential roles of AS in plant response to salt stress.

**Electronic supplementary material:**

The online version of this article (doi:10.1186/1471-2164-15-431) contains supplementary material, which is available to authorized users.

## Background

High salinity in soil is a major environmental condition that adversely affects crop production worldwide. Today, roughly 20% of the world’s cultivated land and nearly half of all irrigated lands are affected by salinity [1]. High concentrations of salt in soil lead to ion imbalances and hyperosmotic stress in plants. Understanding the mechanisms of plant responses to salt stress is fundamentally important to the study of plant biology and also vital to continued development of rational breeding and genetic engineering strategies to improve salt tolerance in crop plants. Plant’s cellular and molecular responses to salt stress have been studied intensively [2, 3]. Among these responses is the dramatic change in the expression of a large number of plant genes, which are regulated at the transcriptional as well as the post-transcriptional levels.

Alternative pre-mRNA splicing (AS) is an important mechanism for regulating gene expression and for increasing transcriptome plasticity and proteome diversity in eukaryotes [4]. AS is involved in many physiological processes in plants, including the response to biotic and abiotic stresses [5–7]. Although AS of some stress-responsive genes has been reported, large-scale or genome-wide studies of AS dynamics under salt stress conditions are still relatively scarce. Based on the data from Sanger sequencing of full-length cDNA libraries from Arabidopsis plants exposed to different stresses such as cold, heat, and salt stress, it was found that the number of AS events under stress conditions (particularly low temperature) was significantly higher than the number under normal conditions [8]. Another study using a whole-genome tiling array in Arabidopsis with various stress treatments identified a group of AS events that were associated with stress-responsive genes and some essential regulatory genes [9]. These and other studies revealed the involvement of AS in response to abiotic stress [7]. The methods used in these studies, Sanger sequencing and tiling array, however suffer from relatively low resolution when compared with the recently developed high-throughput RNA sequencing (RNA-seq) methods. As a result, some AS events, particularly those with lower abundance, may escape detection. A recent study using high-throughput RNA sequencing was conducted with Arabidopsis plants that were exposed to various stresses or were at different developmental stages and time points in the diurnal cycle [10]. That study mainly focused on the complexity of AS rather than on a detailed description of the global changes from AS under salt stress conditions [10].

To investigate the global dynamics of AS under salt stress, in this study, we used the Illumina HiSeq platform to perform pair-end RNA sequencing with Arabidopsis plants that were exposed to different concentrations of salt and generated ~110 million pair-end reads (101 bp in length). In what follows, we first describe the features of AS under salt stress based on comparative AS analysis. We then report on how the genes with differential AS are well associated with specific functional categories, such as the responses to stresses and RNA splicing. We also suggest that AS could represent a regulatory mechanism independent of the regulation of gene transcriptional activation. Finally, we discuss the change in pre-mRNA splicing patterns of serine/arginine-rich (SR) splicing factors under salt stress.

## Results

### Quality analysis of RNA-seq data

**Figure 1:**
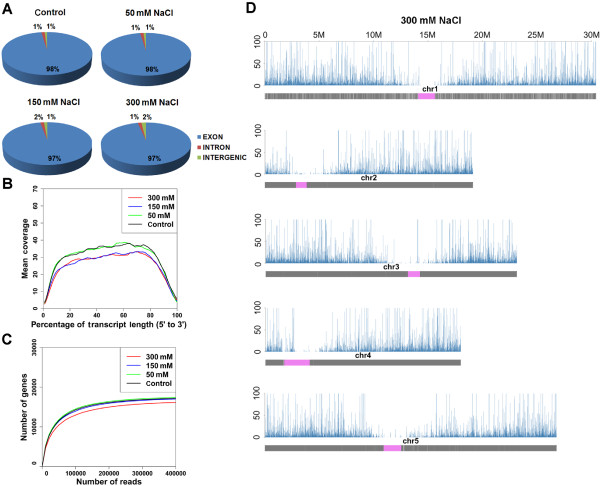
**Quality and features of the RNA-seq datasets obtained in the current study. (A)** Distribution of the RNA-seq reads along annotated Arabidopsis genomic features. Among reads that unambiguously match the Arabidopsis genome, more than 97% reads match annotated exons. **(B)** Relation between the RNA-seq read coverage and the length of the transcriptional unit. The *x*-axis indicates the relative length of the transcripts, and the *y*-axis shows the median depth of coverage. **(C)** Saturation curve for gene detection. Randomly sampled reads are plotted against the expressed genes. The *x*-axis shows the number of the mapped reads and the *y*-axis displays the number of the expressed genes. **(D)** Transcription profiles are plotted across the Arabidopsis genome. The distribution of the RNA-seq read density along the chromosome length is shown. Each vertical blue bar represents log2 of the frequency of reads plotted against the chromosome coordinates. A schematic drawing of the chromosome and its features is shown below the read density. Approximate boundaries of Arabidopsis centromeres are depicted in violet.

We used the mRNA-sequencing (RNA-Seq) method to acquire whole transcriptomes from both NaCl-treated and untreated two-week-old Arabidopsis (ecotype C24) seedlings at the single-nucleotide resolution. To detect salt-induced AS events precisely, we subjected the seedlings to treatments with different concentrations of NaCl (0, 50, 150, or 300 mM). We obtained 110 million sequenced reads (101 bp in length) using the Illumina High-Seq sequencing system. On average, nearly 89% of these reads could be unambiguously aligned to the TAIR10 reference genome sequence (Additional file 1). To evaluate the quality of the RNA-seq data, we investigated the proportion of read alignments in the genome, the continuity of reads (3'/5' bias) along transcriptional units (TUs) and sequencing saturation. Firstly, comparing the mapped reads to the gene annotation revealed that about 98% of the reads were from exonic regions, whereas only 2% were mapped to intergenic and intronic regions (Figure 1A). This was consistent with the quality of the Arabidopsis genome assemblies and annotation. Secondly, plotting the coverage of reads along each transcript exhibited a uniform distribution with no obvious 3'/5' bias, reflecting the high quality of the cDNA libraries (Figure 1B). Lastly, we assessed the sequencing saturation and found that as more reads were obtained, the number of newly discovered genes plateaued (Figure 1C), suggesting that extensive coverage was achieved. This was also supported by plotting the read coverage along each chromosome, which showed extensive transcriptional activity in the entire genome (Figure 1D and Additional file 2). To confirm that the comparison of AS was performed at the same level, we randomly sampled 18 million properly paired mapped reads from each RNA-seq library for further analysis.

### Identification of AS events

To identify AS events, we first predicted splice junctions using the software TopHat, which was designed to identify exon-exon splice junctions. We initially obtained 433,475 junctions from the four RNA-seq libraries (Additional file 3). After filtering the splice junctions by two criteria (for details, see Materials and Methods) - an overhang size of more than 20 bp and at least two reads to support the splice junctions - a junction data set of 397,321 confident junctions that we believe to be true splice junctions was obtained. Comparison of the junctions in this junction data set to the gene annotation (TAIR 10) revealed that about 363,383 (91.5%) junctions were previously annotated, and that the remaining 33,938 (8.5%) were novel junctions that had not been annotated in the TAIR10 Database (Additional file 3).

**Figure 2:**
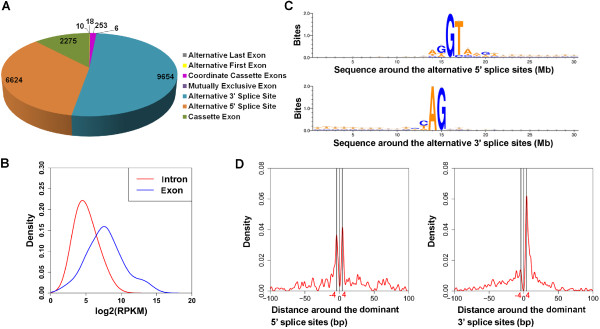
**Features of alternative splicing (AS) in the Arabidopsis genome. (A)** Annotation of AS events based on all the confident junctions. The AS events include cassette exons, alternative 5'SSs, alternative 3'SSs, mutually exclusive exons, coordinate cassette exons, alternative first exons and alternative last exons. **(B)** The distribution of read coverage of the retained introns and the corresponding flanking exons. **(C)** The nucleotide sequences around the alternative 5'SSs and 3’SSs are shown by Weblogo. The results indicate that these alternative 5'SSs and 3'SSs were still associated with GU and AG dinucleotides. **(D)** Distribution of activated alternative 5’SSs and 3’SSs around the dominant ones. These alternative 5'SSs/3’SSs are enriched in the downstream or upstream 4 bp region of the dominant 5'SSs/3’SSs (denoted as position 0 on the *x*-axis). 5'SS, alternative 5' splice site; 3'SS, alternative 3' splice site.

After comparing all the confident junctions to the annotated genes, we identified all the AS events (including 2275 cassette exons, 6624 alternative 5' splice sites (SSs), 9654 alternative 3'SSs, 6 mutually exclusive exons, 253 coordinate cassette exons, 18 alternative first exons and 10 alternative last exons) under salt stress (Figure 2A). We also identified 35,565 intron retention events that had at least five intron-reads (i.e., the reads were mapped within introns) and more than 80% of the intron region covered by intron-reads. Among all these AS events, 45.3% had already been annotated in Arabidopsis genes (TAIR10), and the remaining 54.7% were identified as novel AS events. Based on all identified events, we found that about 49.4% of intron-containing genes were alternatively spliced under salt stress.

Intron retention was the most prevalent AS event under salt stress, although most intron retentions had relatively low read coverage compared to the read coverage of exons (Figure 2B). This is consistent with the intron-retention background in Arabidopsis that was recently reported [11]. Following intron retention, the alternative 5' and 3' splice sites were relatively prevalent compared with the other types of AS events. Sequence analysis of alternative 5' splice sites (5'SSs) and alternative 3' splice sites (3'SSs) revealed that these activated splice sites were still associated with GU and AG dinucleotides (Figure 2C). Moreover, we found that the occurrence of these alternative 5'SSs and 3'SSs was enriched in the downstream and upstream 4 bp region of the dominant 5'SSs and 3'SSs (Figure 2D), respectively. These features of alternative 5'SSs and 3'SSs are consistent with those found in the human genome [12]. It is noteworthy that when correlating exon skipping events to alternative 5'SSs and 3'SSs, we found that about ~17% of the skipped exons simultaneously had alternative 5'SSs or 3'SSs. This percentage of occurrence was significantly higher than that expected for random sampling of all annotated exons (the probability of random occurrence is 0.02%, Fisher Exact Test, *p* < 0.001). This result suggests a coordinated occurrence of exon-skipping and alternative splice site selection.

### Salt-stress enhances AS

**Figure 3:**
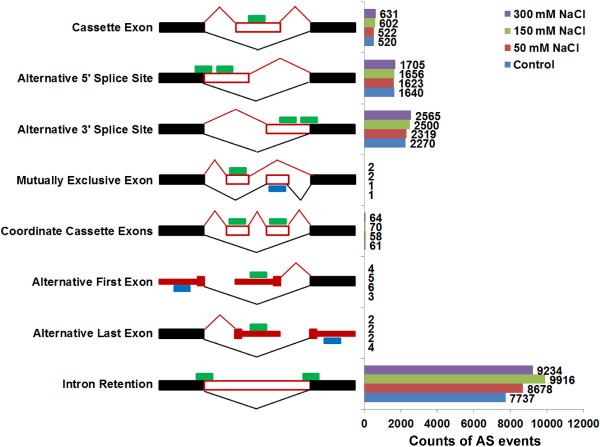
**The counts of each type of AS events in the control and 50, 150 or 300 mM NaCl treatments.** The number of alternative 5'SSs, 3’SSs and exon-skipping events are more in the NaCl treatments than in the control treatment. The green/blue bars represent forward and reverse sequencing reads. 5'SS, alternative 5' splice site; 3'SS, alternative 3' splice site.

We next compared the difference in AS between the control and NaCl treatments. We found that the number of AS events in salt-treated plants was obviously higher than that in the control plants (Figure 3), consistent with a previous report [8]. We ran a Fisher’s Exact Test on the junction-read-counts/intron-read-counts (only for intron retention) and the corresponding exon-read-counts between the control and the treatments and identified 2065 AS events (including 279 alternative 5'SSs, 486 alternative 3'SSs, 102 exon-skipping, and 1198 intron retention events) from 1088 genes that were significantly over-represented in NaCl-treated plants (Additional files 4, 5, 6, 7 and 8). In contrast, we identified only 1320 AS events (including 184 alternative 5'SSs, 247 alternative 3'SSs, 53 exon-skipping, and 836 intron retention events) from 643 genes that were absent from these NaCl-treated plants (Additional files 4, 9, 10, 11 and 12). These data indicated the overall promotion of AS by salt stress.

### Changes in splicing patterns associated with stress response

**Figure 4:**
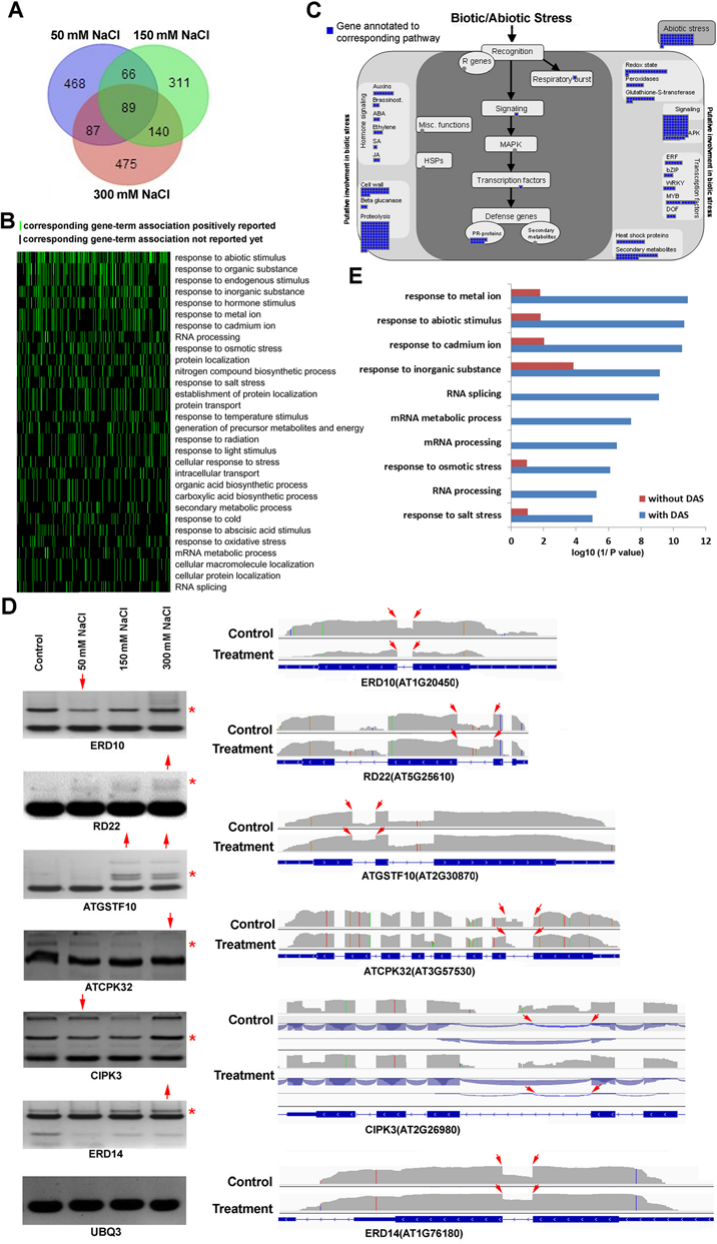
**Genes with abnormal splicing in NaCl treatments are closely associated with stress response and transcriptional activation. (A)** Venn diagram of the overlap of differential alternative splicing (DAS) genes in 50, 150 or 300 mM NaCl treatments. **(B)** A two-dimensional (2-D) view of the relationship between the genes with abnormal splicing and their functional annotations. The functional classification of genes was done by the DAVID software. Top 30 of the functional annotations that were ordered by the enrichment scores were selected for the 2-D view. **(C)** A network generated by Mapman indicates that genes with aberrant splicing are involved in various stress response pathways, including hormone-signaling pathways, MAPK-signaling pathways and transcription regulation. **(D)** The representative AS events in six stress-responsive genes validated by RT-PCR and visualized by IGV browser. In the RT-PCR validation, the red asterisk (*) to the right side denotes the alternative splice form. The red arrow on the top indicates an increase (pointing upward) or decrease (pointing down) in AS events at that salt concentration. In the IGV visualization, the exon-intron structure of each gene is given at the bottom of each panel. The grey peaks above the exon-intron structure indicate the RNA-seq read density across the gene. The red arrows represent alternative splice sites. The blue arcs in *CIPK3* indicate splice junction reads that support the junctions. **(E)** Enrichment of biological processes in DAS genes and genes without DAS. The top 10 functional categories in DAS genes are shown. In the stress-related and RNA processing categories, the possibility of genes that undergo AS is much higher than the possibility of genes that are not alternatively spliced.

To investigate the potential influence of salt-stress-induced AS on cellular processes, we analyzed functional categories and pathways of the genes with differential AS under salt stress. We identified 1636 differential alternative splicing (DAS) genes in seedlings treated with 50, 150 or 300 mM NaCl, of which 28.3% were found in the seedlings from at least two of these treatments (Figure 4A). An analysis of functional categories using the software DAVID [13, 14] revealed that these differentially spliced genes were involved in several biological processes, including responses to abiotic stimulus and RNA processing, suggesting that salt stress may impact biological processes through changing pre-mRNA splicing (Additional file 13). In particular, the response-to-abiotic-stimulus functional category was markedly increased among the DAS genes, and was observed in the seedlings in all the salt stress treatments (Figure 4B and Additional file 14). The results suggested that AS under salt stress was not a random process. Rather, it was associated with the stress response. Indeed, further analysis using Mapman [15] suggested that genes with aberrant splicing in NaCl-treated seedlings were involved in various stress response pathways, including hormone-signaling pathways, MAPK-signaling pathways, and transcription regulation of stress responses (Figure 4C and Additional file 15). Notably, some important genes (such as *ERD10*, *RD22, ATGSTF10*, *ATCPK32*, *CIPK3* and *ERD14*) involved in stress responses were differentially alternatively spliced in the NaCl-treated plants (Figure 4D). Among them, *ATCPK32* is an ABA signaling component that regulates the ABA-responsive gene expression via ABF4 [16], and ERD10 encodes a gene induced by low temperature and dehydrations [17]. Both genes showed decreased retention of their first introns under salt stress. In contrast, the other three genes (*ERD14*, *RD22*, and *ATGSTF10*) involved in abiotic stress responses [18–20] showed increased intron retention under salt stress. These intron retention events were validated by RT-PCR using intron-flanking primers. The amount of the corresponding PCR products was either increased or decreased under salt stress, consistent with the RNA-seq data.

Sequence analysis of these intron-retained transcripts suggested that all of these intron retentions could generate pre-mature stop codons. Therefore, the decrease or increase in intron retention was predicted to increase or decrease the abundance of the functional transcripts, respectively. Since these genes and many other genes with significant intron retentions (Additional files 8 and 12) have been suggested to play roles in stress responses and they are induced by abiotic stresses, an increase in their functional transcript levels (e.g., *ATCPK32* and *ERD10*) is likely to have positive effects on salt tolerance, whereas a decrease in the functional transcript levels of other genes (e.g., *ERD14*, *RD22* and *ATGSTF10*) could have negative effects on salt tolerance in plants. These results suggested that alteration of AS in stress-responsive genes might impact a plant’s tolerance to salt stress.

We further compared the functional categories of DAS genes with the functional categories of genes without DAS. This comparison clearly revealed that different functional categories are over-represented in both populations (Additional files 13 and 16). Generally, among genes that produce alternative transcripts, several Gene Ontology (GO) categories related to stress, such as ‘response to metal ion’ , ‘response to abiotic stimulus’ , ‘response to cadmium ion’ , ‘RNA splicing’ and ‘RNA processing’ , are over-represented. On the other hand, among genes that are not alternatively spliced, several functions related to house-keeping, such as ‘protein transport’ , ‘DNA repair’ and ‘cell wall organization’ , are over-represented. The presence of genes that undergo AS in the stress-related and RNA processing categories is much higher than the presence of genes not alternatively spliced in these categories, further supporting the notion that stress-related genes are more predisposed to pre-mRNA processing than are genes involved in basic cellular functions (Figure 4E).

### AS and gene expression are separately regulated in response to salt stress

**Figure 5:**
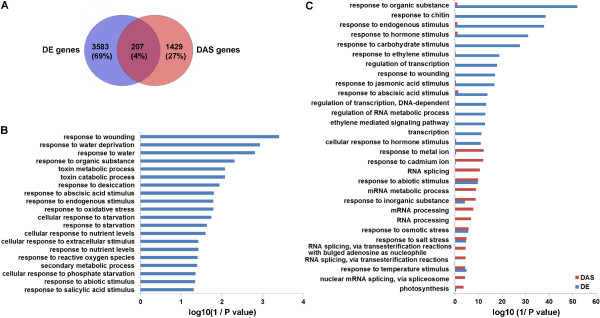
**Functional categorization of differentially expressed (DE) genes and differential alternative splicing (DAS) genes. (A)** Venn diagram of the overlap of DE and DAS genes. **(B)** Functional categorization (biological process) of 207 DE genes that are also DAS genes. **(C)** Functional categorization (biological process) of genes with only DE or DAS.

From the RNA-seq data, 1,368, 1,901 and 2,729 genes were defined as differentially expressed (DE) in the 50, 150 or 300 mM NaCl treatments, respectively, relative to the control (*p* < 0.01, or fold change >2 and *p* < 0.05) (Additional file 17). The differentially expressed genes identified in our study overlapped with those identified by other groups based on Microarray analyses of salt-stressed Arabidopsis seedlings (data from the Genevestigator database), indicating that the salt-stress-induced gene regulation found here was comparable to that of other studies (Additional file 18). Interestingly, when compared with the DAS genes, only 207 DE genes also exhibited significantly changed intron retention, alternative 5’SSs, alternative 3’SSs or exon skipping under NaCl treatments (Figure 5A). Functional categorization of these 207 genes revealed that they are enriched in several important functional pathways, such as ‘response to abiotic stimulus’ (Figure 5B), indicating that a subset of genes with critical salt stress response functions are regulated both transcriptionally and post-transcriptionally.

Nonetheless, these co-regulated genes account only for a relatively small portion of all DAS or DE genes in Arabidopsis. This finding suggested that AS and gene activation could be separately regulated in response to salt stress. Indeed, analysis of the DAS and DE genes confirmed that the over-represented functional categories differed largely between the two groups, revealing separate regulation of gene expression and AS in response to salt stress (Additional files 19 and 20). For example, some functional categories, e.g., ‘RNA splicing’ and ‘RNA processing’ , were over-represented only among DAS genes, while other categories, such as ‘transcription’ and ‘response to hormone stimulus’ , were found among the DE genes (Figure 5C).

### Frequent alteration of SR splicing factors in AS patterns under salt stresses

Whereas genes involved in RNA splicing are mostly not regulated by salt stress at the expression level, these genes are frequently alternatively spliced under salt stress. AS of these splicing-related genes could therefore represent an independent means of regulation of genes in response to salt stress. Strikingly, we identified 15 splicing factors with changes in AS under salt stress (Additional file 21). Ten of these splicing factors encoded SR (serine/arginine-rich) proteins. SR splicing factors play key roles in the execution and regulation of pre-mRNA splicing in plants. In Arabidopsis, there are a total of 18 SR proteins [21, 22]. Previous studies suggested that pre-mRNAs of SR protein genes were frequently alternatively spliced under environmental stress, which is thought to alter the splicing of their targets and result in adaptive transcriptome changes in response to environmental conditions [23, 24].

**Figure 6:**
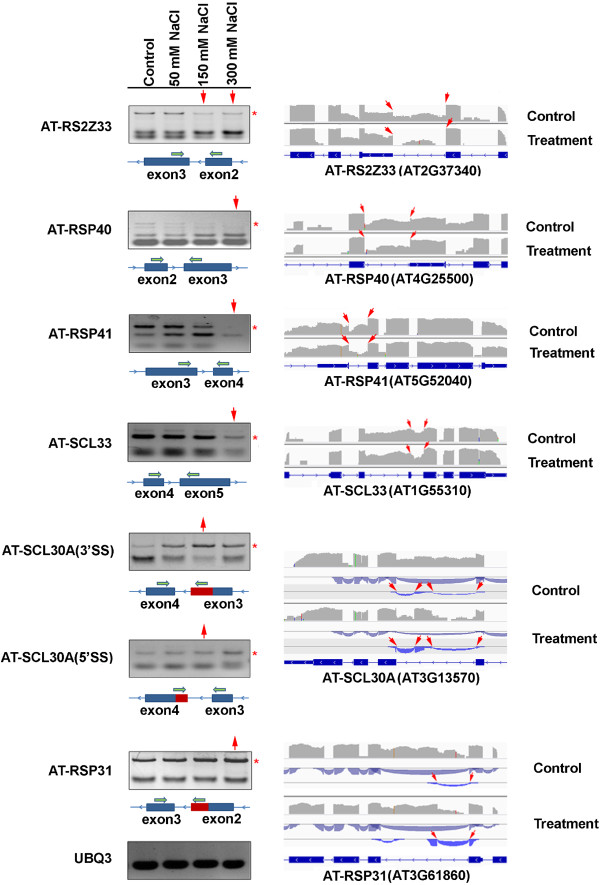
**Eight AS events in six SR genes validated by RT-PCR and visualized by the IGV browser.** In the RT-PCR validation, the grey asterisk (*) to the right side denotes the alternative splice form. The red arrow on top indicates that an increase (pointing upward) or decrease (pointing down) in AS events is exhibited at that salt concentration. In the schematic exon-intron structure below each gel picture, the blue bars represent exons and the red bars represent splice junctions. The green arrows indicate primers designed for RT-PCR validation. In the IGV visualization, the exon-intron structure of each gene is given at the bottom of each panel. The grey peaks above the exon-intron structure indicate the RNA-seq read density across the gene. The red arrows represent alternative splice sites. The blue arcs indicate splice junction reads that support the junctions. 5'SS, alternative 5' splice site; 3'SS, alternative 3' splice site.

We validated six of all these splicing factors by RT-PCR and visualized them using the IGV junction browser (Figure 6). Among the visualized genes, four SR genes (*AT-RSP40*, *AT-RSP41*, *AT-RS2Z33* and *AT-SCL33*) exhibited a decrease in AS events under salt stress, and two SR genes (*AT-RSP31* and *AT-SCL30A*) exhibited an increase in AS events under salt stress (Figure 6). The intron retentions of *AT-RS2Z33* and *AT-RSP40* were detected in the second intron in plants under the control conditions, but they were weakly present in the plants treated with NaCl. This was also verified by RT-PCR using a forward primer in the third exon and a reverse primer in the second exon. The intron retention in both genes occurred in the 5’UTR region, which would lead to abnormal transcripts with long 5’UTRs that could interrupt the translation and lead to reduced synthesis of the protein. The decreased intron retention in these two genes under salt stress should therefore lead to a decrease in the level of these corresponding long 5’UTR transcripts, which could consequently increase the abundance of their functional transcripts and proteins. The intron retention of genes *AT-RSP41* and *AT-SCL33* that respectively occurred in the third and fourth introns were clearly detected in the control, while they were barely present in samples treated with 300 mM NaCl treatment. This was validated by RT-PCR using intron-flanking primers. Sequence analysis revealed that both intron retention events introduced premature stop codons (PTCs) and generated truncated proteins. Therefore, the decreased intron retention in both genes under salt stress could lead to the decrease in abnormal transcripts and the increase in functional transcripts.

Alternative 3'SS and 5'SS were found in the third intron of *AT-SCL30A* (AT3G13570) under the 150 mM NaCl treatment, while they were weakly present in the control. This observation was validated by RT-PCR using a forward primer in the third/fourth exon and a reverse one covering the splice junction (Figure 6). Further detailed analysis revealed that both alternative 3'SS and 5'SS actually introduced a novel exon (not annotated in the TAIR10 Arabidopsis genome) that was inserted into the region between the third and fourth exon and generated a novel isoform. Sequence analysis for this isoform suggested that this exon insertion would generate PTCs and thus could encode a truncated protein that was composed of 120 amino acids. Therefore, this exon insertion under salt stress could lead to a decrease in the functional transcripts. Nonetheless, it is unclear whether this novel isoform has any function.

Finally, we identified an alternative 3'SS in the second intron in *AT-RSP31*, with an increased level under the 300 mM NaCl treatment. This observation was validated by RT-PCR using a forward primer covering the splice junction and a reverse one in the second exon (Figure 6). This alternative 3'SS (not annotated in the TAIR10 Arabidopsis genome) extends the third exon into the next intron and thus generates a larger exon. Sequence analysis for this isoform suggested that this alternative 3'SS would introduce PTCs and thus could encode a truncated protein. It is also unclear whether this truncated protein has any function.

## Discussion

Through comprehensive transcriptome analysis of high-throughput RNA-seq data, in this study, we disclosed features of genome-wide AS in Arabidopsis under salt stress. Our analysis suggests that 49% of the intron-containing genes in Arabidopsis genome are alternatively spliced under salt-stress conditions. Moreover, we found that AS is increased by salt stress and that 10% of the intron-containing genes showed significantly differential AS under salt-stress conditions. The analysis of functional categories demonstrated that genes with differential AS are associated with responses to stress and RNA splicing. Finally, we observed that genes encoding the splicing factors, i.e., SR proteins, are subject to frequent and specific AS under salt stress.

### An overview of AS in Arabidopsis under salt stress

Recent studies using massively parallel RNA sequencing revealed that a large percentage of genes in Arabidopsis undergo AS [10, 11], which potentially could significantly increase the plasticity of the transcriptome and proteome diversity. In this study, we conducted systematic analysis of the transcriptome of salt-treated Arabidopsis plants. Our data revealed that, under salt stress, 49% of the intron-containing genes are alternatively spliced. This number is higher than that reported by Filichkin et al. (42%) [10], but very close to that reported by Li et al. (48%) [25], and lower than a recent report that 61% of multi-exonic genes were alternatively spliced, as determined by a normalized cDNA library that facilitated the detection of AS events in low-abundance transcripts [11]. This marked AS under salt stress could provide molecular plasticity for the plants to adapt to stress conditions.

In this study, we found that intron retention and alternative 5’SSs/3’SSs are much more prominent than exon skipping and other types of AS. These observations are consistent with the general view of AS in Arabidopsis reported previously [10, 11]. Importantly, we uncovered two novel features of AS in Arabidopsis. First, alternative 5’SSs/3’SSs tend to occur around the downstream or upstream 4 bp region of the dominant (conical) 5'SS and 3'SS (Figure 2D). A similar AS pattern was also reported in the human genome [12], suggesting the conservation of this AS pattern in eukaryotes. Second, we found a coordinated occurrence of exon skipping and alternative splice site selection. We thus proposed a model where exon skipping and alternative splice site selection are coupled. We suggest that all the splice sites surrounding the dominant ones have the potential to be used as alternative splice sites. These include the splice sites located at the next or last exon, which would thus cause exon skipping. Previously, exon skipping and alternative splice site selection were usually considered as two independent AS events. Few links between them were previously reported. The discovery of the linkage between these two AS events provides a novel perspective of AS and its regulation.

### Are stress-induced changes in splicing patterns stress-associated acclimation or damage?

We found that AS events were obviously increased in Arabidopsis under salt stress. This finding is consistent with some previous studies on AS under environmental stresses [5]. For example, cDNA sequencing results indicated that the number of AS events was significantly higher in Arabidopsis plants exposed to different stresses, particularly low temperature, than in control plants [8]. This increased AS under stress conditions raises an important question on whether the increase is an acclimation response or merely a consequence of splicing errors caused by stress damage. We tend to believe that the increase comes from splicing errors based on the following reasons.

First, in another study on the effects of the depletion and overexpression of one core component of the splicing machinery (SAD1, a Sm-like protein 5) on pre-mRNA splicing and stress tolerance [26], we found that the increase or decrease of AS in many stress-related genes can be dynamically controlled by the dosage of SAD1; moreover, the increase and decrease in AS are closely linked to the sensitivity and tolerance of the plants to stress, respectively. Therefore, we considered that increased AS could be a result of inaccurate splicing, which could weaken the function of the corresponding genes by decreasing the functional transcripts. In contrast, decreased AS could be an acclimation to stress resistance. Secondly, we did observe a stress-induced deregulation of splicing machinery. In our study, we noticed the down-regulation of U6 snRNAs under salt stress in quantitative RT-PCR assays (Additional file 22). The U6 snRNA is a core component of the spliceosome and is required for its assembly and catalytic activity during pre-mRNA splicing [27, 28]. A decrease in the level of this snRNA would likely compromise the assembly of the spliceosome and its catalytic activity [29]. Thirdly, most stress-induced splicing variants may not be translated into functional proteins. Similarly, some important genes (such as from *ERD14*, *RD22* and *ATGSTF10*) that are involved in abscisic acid (ABA) or salt stress responses show increased intron retention under salt stress conditions. These transcripts were predicted to generate a pre-mature stop codon that would lead to non-functional mRNAs or proteins, although we currently could not rule out the possibility that some of these truncated proteins may still have certain functions in plant salt tolerance. Thus, we suggest that stress-induced increase in AS could be ascribed to splicing errors or inaccuracies caused by stress.

Nevertheless, if the increase in AS is merely a non-specific consequence of stress damage, a random distribution would be expected among genes. However, our data, along with previous reports, demonstrated that the genes associated with stress response tend to be alternatively spliced under stress conditions (Figure 4B). It is known that salt stress or other abiotic stresses can activate the expression of a large number of plant stress-responsive genes that are not expressed or are expressed at lower levels under normal non-stressful conditions [30, 31]. With the simultaneous production of a large amount of these stress-inducible pre-mRNAs, cells would need to immediately recruit a significant amount of splicing factors and other factors for their co-transcriptional or post-transcriptional processing. This imposes a huge burden on the splicing machinery and, as a result, a significant portion of these transcripts fails to be processed adequately when the splicing machinery is compromised.

The discussion so far covers only to the global changes in AS under salt stress conditions. It should be noted that there are indeed specific cases in which AS plays a functional role in regulating the response and tolerance of plants to stress. Such cases have been described in the last few years [review in [5]. This functional role can also be seen in the splicing of several SR proteins as discussed below.

### Pre-mRNA spicing of SR genes under stress conditions

The AS pattern of several SR proteins has been shown to change obviously under various abiotic stress conditions, including temperature stress, high salinity and high light irradiation [21, 32, 33]. In this study, we identified one- third of the SR genes (six SR genes from four SR families) that showed clear changes in AS under salt stress. This number is higher than that reported before and is probably attributable to the increased sensitivity of the sequencing technology used in the current study.

Interestingly, we clearly identified four SR genes (*AT-RS2Z33*, *AT-RSP40*, *AT-SCL33* and *AT-RSP41*) that showed decreased intron retention under salt stress (Figure 6). Sequence analysis revealed that all the splice variants with reduced abundance under salt stress were aberrant transcripts with premature stop codons that may not produce functional proteins. A decrease in these aberrant transcripts and a simultaneous increase in the functional transcripts in these SR genes could be an acclimation response to stress that may subsequently help to sustain a positive feedback loop to increase the splicing efficiency and the production of functional proteins to combat the stress. Consistently, our recent study demonstrated that the mutations of *AT-RSP40* or *At-RSP41* led to sensitivity to salt stress, which implied the positive role of *At-Rsp40* or *At-Rsp41* in salt stress tolerance, probably via regulating the pre-mRNA splicing of certain stress tolerance genes [34]. We predict that regulating the expression of some of these SR genes or other splicing factors may increase plant tolerance to salt stress by enhancing the correct splicing of salt tolerance genes. Our recent study [26] and a few other studies showed that over-expression of certain splicing factors indeed could increase plant tolerance to salt and other stresses [21, 32, 33].

## Conclusions

Through analyzing global changes in AS under salt stress, we firstly identified ~49% of all intron-containing genes that were alternatively spliced under salt stress, 10% of which experienced significant differential alternative splicing (DAS). We found that most DAS genes were not differentially regulated by salt stress, suggesting that AS may represent an independent layer of gene regulation in response to stress; DAS genes were associated with specific functional pathways, such as the pathways for the responses to stresses and RNA splicing. Finally, we revealed that serine/arginine-rich (SR) splicing factors were frequently and specifically regulated in AS under salt stresses, suggesting a complex loop in AS regulation for stress adaptation. Therefore, our study provided a comprehensive view of AS under salt stress and revealed novel insights into the potential roles of AS in plant response to salt stress.

## Methods

### Plant materials and growth conditions

Seeds of the Arabidopsis (ecotype C24) plants were surface-sterilized with 50% bleach in 0.01% Triton X-100 and planted on ½ Murashige and Skoog (MS) medium agar plates supplemented with 3% sucrose. After 4-day stratification at 4°C, the plates were placed in a chamber (Model CU36-L5, Percival Scientific, Perry, IA, USA) under 16 h-white light (~75 μmol m^−2^ s^−1^) and 8 h-dark conditions at 21 ± 1°C for germination and seedling growth. Twelve days after being incubated at 21 ± 1°C in the chamber, twenty whole seedlings were transferred from the agar plate onto filter paper (Catalog No. 05-714-4, Fisher Scientific, Pittsburgh, PA, USA) saturated with 20 ml of 0, 50, 150, or 300 mM NaCl solution in a 150 × 15 mm petri dish and incubated in the same chamber for 3 h before being harvested and frozen in liquid nitrogen for total RNA extraction [35].

### RNA extraction, library construction and sequencing

Using the TRIzol Reagent (Catalog No. 15596–026, Invitrogen), total RNAs were extracted from seedlings without or with salt stress treatment. Polyadenylated RNAs were isolated using the Oligotex mRNA Midi Kit (Catalog No. 70042, Qiagen). The RNA-seq libraries were constructed using the Illumina Whole Transcriptome Analysis Kit following the standard protocol (Illumina, HiSeq system) and sequenced by the Bioscience Core Facility at KAUST on the HiSeq platform to generate high-quality pair-end reads.

### Reads alignment and junction prediction

TopHat [36] was used to align the reads against the Arabidopsis genome sequences and annotated gene models downloaded from TAIR10 (http://www.arabidopsis.org/) with default parameters. Meanwhile, TopHat was also used to predict the splice junctions. Based on the gene annotation information, the splice junctions were classified into the known and novel splice junctions. In addition, the expressed gene or transcripts were identified by the Cufflinks software [37].

### Determination of the criteria for filtering positive junctions

In the initial prediction, there were a great number of novel junctions that had short overhangs (i.e., fewer than 20 bp) with the corresponding exons, while most of the annotated junctions had larger overhangs, with the enrichment at ~90 bp (Additional file 23A). Moreover, the novel junctions had relatively low coverage compared to the annotated junctions (Additional file 23B). In general, the junctions with short overhang size and lower coverage were considered as false positives, which are often caused by non-specific or error alignment. Therefore, to distinguish between true splice junctions and false positives, we assessed the criteria based on simulating data on a set of randomly-constituted junctions. To do this, we firstly generated a set of 80,000 splice junctions in which annotated exons from different chromosomes were randomly selected and spliced together *in silico*, and 119,618 annotated junctions from the gene annotation. Since the length of our sequencing reads was 101 bp, the splice junction sequences were determined to be 180 bp long (90 nt on either side of the splice junction) to ensure a 11 bp overhang of the read mapping from one side of the junction onto the other. Alignments to the random splice junctions were considered to be false positives, because such junctions are thought to rarely exist when compared to annotated junctions. The alignments of the raw RNA-seq reads to the random junctions revealed that 99.9% of false positive junctions had overhang sizes with fewer than 20 bp. In sharp contrast, the alignments of the annotated junctions indicated that most (98.6%) of annotated junctions had larger overhang sizes (Additional file 24A). In addition, we estimated that 56.9% of false positive junctions had only one read spanning the junction, while the annotated junctions had higher read coverage (Additional file 24B). To minimize the false positive rate, we required that the overhang size had to be greater than 20 bp with at least two reads spanning the junctions. Using these criteria, we filtered out almost all false positive junctions (Additional file 24C).

### Annotation of AS events

JuncBASE [38] was used for annotating all AS events, including cassette exons, alternative 5'SSs, alternative 3'SSs, mutually exclusive exons, coordinate cassette exons, alternative first exons, alternative last exons and intron retention, which takes as input the genome coordinates of all annotated exons and all confidently identified splice junctions.

### Global comparison of AS

The global comparison of AS among the control (0), 50, 150 or 300 mM NaCl treatments was started by equally and randomly re-sampling uniquely mapped reads to make sure that the comparison was at the same level. The comparison refers to the two facets: the absolute number of each type of AS event and the number of junction reads that was assigned to each type of AS event, because both of them can be used to measure the global changes of AS. Meanwhile, Fisher’s Exact Tests in R (http://www.r-project.org/) were used to identify differential representation of each type of AS event, performed on the number of junction reads that were assigned to each type of AS event.

### The identification of differential AS events

Fisher’s Exact Tests were also used to identify the differential representation of each AS event. For alternative 5'SSs and 3'SSs and exon skipping events, Fisher’s Exact Tests were performed on the comparison of the junction-read counts and the corresponding exon-read counts between the control and the 50, 150 or 300 mM NaCl treatments. The events with *p* values less than 0.05 were identified as significantly differential events. In addition, we considered those AS events that were uniquely identified in the control or the 50, 150 or 300 mM NaCl treatments significant if there were at least five junction reads to support and if the *p* value of these events was assigned to equal zero. Similarly, for intron retention, Fisher’s Exact Tests were performed on the intron-read counts and the corresponding exon-read counts between the control and the 50, 150 or 300 mM NaCl treatments. The events with *p* value less than 0.001 were identified as significant differential events. In addition, we considered the intron retention events uniquely identified in the control or the 50, 150 or 300 mM NaCl treatments significant if there were at least 5 × sequence coverage to support and if more than 80% of intron regions were covered by intron-reads. The *p* value of these events was assigned to equal zero.

### RT-PCR validation

The selected AS and intron retention events were validated by RT-PCR using a set of primers (Additional file 25) that were designed based on each AS event. Total RNAs from the control, 50, 150 or 300 mM NaCl treated seedlings were extracted as described above using the TRI solution, treated with DNAase I, and reverse-transcribed to cDNA (random priming) by using a standard protocol (SuperScript II reverse transcriptase, Invitrogen).

## Availability of supporting data

We deposited the RNA-seq data in the Sequence Read Archive (SRA) database with an accession number SRP035234.

## Electronic supplementary material

Additional file 1: **Mapping result of RNA-seq reads.** (XLSX 10 KB)

Additional file 2: **Transcription profiles in the control, 50 or 150 NaCl treatments were plotted across the Arabidopsis genome.** Distribution of the RNA-seq read density along the chromosome length is shown. Each vertical blue bar represents log2 of the frequency of reads plotted against the chromosome coordinates. A schematic drawing of the chromosome and its features is shown below the read density. Approximate boundaries of Arabidopsis centromeres are depicted in violet. (PDF 954 KB)

Additional file 3: **Total junctions from four RNA-seq libraries.** (XLSX 10 KB)

Additional file 4: **The number of DAS events in 50, 150 or 300 mM NaCl treatments.** Blue bars indicate the number of DAS events that are significantly over-represented in NaCl treatment plants. Red bars indicate the number of DAS events that are absent in NaCl treatment plants. (PDF 179 KB)

Additional file 5: **List of alternative 5' splice sites that were over-represented in the 50, 150 or 300 mM NaCl treatments.** (XLSX 33 KB)

Additional file 6: **List of alternative 3' splice sites that were over-represented in the 50, 150 or 300 mM NaCl treatments.** (XLSX 50 KB)

Additional file 7: **List of exon-skipping events that were over-represented in the 50 mM, 150 mM or 300 mM NaCl treatments.** (XLSX 17 KB)

Additional file 8: **List of genes with intron retention in the 50, 150 or 300 mM NaCl treatments.** (XLSX 117 KB)

Additional file 9: **List of alternative 5' splice sites that were over-represented in the control.** (XLSX 25 KB)

Additional file 10: **List of alternative 3' splice sites that were over-represented in the control.** (XLSX 30 KB)

Additional file 11: **List of exon-skipping events that were over-represented in the control.** (XLSX 13 KB)

Additional file 12: **List of genes with intron retention in the control.** (XLSX 83 KB)

Additional file 13: **Top 50 of the functional categorization (biological process) of DAS genes.** (XLSX 34 KB)

Additional file 14: **A two-dimensional (2-D) view of the relationship between the genes with abnormal splicing and their functional annotations in 50, 150 or 300 mM NaCl treatments.** The functional classification of genes was done by the DAVID software. The top 20 functional annotations that were ordered by the enrichment scores were selected for the 2-D view, which indicates that genes with abnormal splicing were strikingly enriched in the response-to-abiotic-stress category. (PDF 2 MB)

Additional file 15: **A network generated by Mapman indicates that genes with aberrant splicing in the 50, 150 or 300 mM NaCl treatments were involved in various stress response pathways, including hormone-signaling pathways, MAPK-signaling pathways and transcription regulation.** (PDF 740 KB)

Additional file 16: **Top 50 of the functional categorization (biological process) of genes without DAS.** (XLSX 498 KB)

Additional file 17: **List of differentially expressed genes in the 50, 150 or 300 mM NaCl treatments relative to the control.** (XLSX 696 KB)

Additional file 18: **The heatmaps generated by mapping the DE genes in the 50, 150 or 300 mM NaCl treatments to the database of microarray data with Genevestigator.** The heatmaps indicate that DE genes (including the up-regulated and down-regulated genes) in NaCl treatments are consistent with the gene profiles derived from the microarray data. (PDF 8 MB)

Additional file 19: **Top 50 of the functional categorization (biological process) of DE genes.** (XLSX 108 KB)

Additional file 20: **Top 50 of the functional categorization (biological process) of DAS genes.** (XLSX 159 KB)

Additional file 21: **The list of identified SR genes with DAS in this study.** (XLSX 26 KB)

Additional file 22: ***U6*** **snRNA expression levels as determined by quantitative RT-PCR.** The snRNA level in NaCl treatment plants was lower than in the control plants. (PDF 50 KB)

Additional file 23: **The distinctive features between known and novel splice junctions.** **(A)** The density of the overhang size with exons for known and novel splice junctions in each sample. The *x*-axis indicates the size of the overhang with exon and the *y*-axis indicates the density of the sizes. A great number of novel junctions has shorter overhangs (i.e., fewer than 20 bp) with the corresponding exons, while most of the annotated junctions have larger overhang size, with the enrichment at ~90 bp. **(B)** The density of junction-read coverage for known and novel junctions. The novel junctions have relatively low coverage compared to the annotated junctions. (PDF 382 KB)

Additional file 24: **The features of false positive (random) and annotated junctions.** **(A)** The density of the overhang size of false positive and annotated junctions. Most of false positive junctions have shorter overhang sizes, while the annotated junctions have larger overhang sizes. **(B)** The density of junction-read coverage of false positives and annotated junctions. More than half of false positive junctions have only one read spanning the junction, while the annotated junctions have higher reads coverage. **(C)** Distinguishing true junctions from false positive alignments. To reduce the number of false positive junctions, as determined by randomly generated junctions, we required that the overhang size must be more than 20 bp with at least two reads spanning the junctions. Using both criteria, the false positive junctions sharply reduced to very low levels (close to zero). In contrast, the annotated junctions show no obvious decrease. (PDF 552 KB)

Additional file 25: **The primers used for RT-PCR validation.** (XLSX 50 KB)

## References

[1] Zhu JK (2002). Salt and drought stress signal transduction in plants. Annu Rev Plant Biol.

[2] Hasegawa PM, Bressan RA, Zhu JK, Bohnert HJ (2000). Plant cellular and molecular responses to high salinity. Annu Rev Plant Physiol Plant Mol Biol.

[3] Munns R, Tester M (2008). Mechanisms of salinity tolerance. Annu Rev Plant Biol.

[4] Chen M, Manley JL (2009). Mechanisms of alternative splicing regulation: insights from molecular and genomics approaches. Nat Rev Mol Cell Biol.

[5] Mastrangelo AM, Marone D, Laido G, De Leonardis AM, De Vita P (2012). Alternative splicing: enhancing ability to cope with stress via transcriptome plasticity. Plant Sci.

[6] Reddy AS, Marquez Y, Kalyna M, Barta A (2013). Complexity of the alternative splicing landscape in plants. Plant Cell.

[7] Staiger D, Brown JW (2013). Alternative splicing at the intersection of biological timing, development, and stress responses. Plant Cell.

[8] Iida K, Seki M, Sakurai T, Satou M, Akiyama K, Toyoda T, Konagaya A, Shinozaki K (2004). Genome-wide analysis of alternative pre-mRNA splicing in *Arabidopsis thaliana* based on full-length cDNA sequences. Nucleic Acids Res.

[9] Eichner J, Zeller G, Laubinger S, Ratsch G (2011). Support vector machines-based identification of alternative splicing in *Arabidopsis thaliana* from whole-genome tiling arrays. BMC Bioinformatics.

[10] Filichkin SA, Priest HD, Givan SA, Shen R, Bryant DW, Fox SE, Wong WK, Mockler TC (2010). Genome-wide mapping of alternative splicing in *Arabidopsis thaliana*. Genome Res.

[11] Marquez Y, Brown JW, Simpson C, Barta A, Kalyna M (2012). Transcriptome survey reveals increased complexity of the alternative splicing landscape in Arabidopsis. Genome Res.

[12] Roca X, Sachidanandam R, Krainer AR (2005). Determinants of the inherent strength of human 5' splice sites. RNA.

[13] Huang DW, Sherman BT, Lempicki RA (2009). Systematic and integrative analysis of large gene lists using DAVID bioinformatics resources. Nat Protoc.

[14] Huang DW, Sherman BT, Lempicki RA (2009). Bioinformatics enrichment tools: paths toward the comprehensive functional analysis of large gene lists. Nucleic Acids Res.

[15] Thimm O, Blasing O, Gibon Y, Nagel A, Meyer S, Kruger P, Selbig J, Muller LA, Rhee SY, Stitt M (2004). MAPMAN: a user-driven tool to display genomics data sets onto diagrams of metabolic pathways and other biological processes. Plant J.

[16] Choi HI, Park HJ, Park JH, Kim S, Im MY, Seo HH, Kim YW, Hwang I, Kim SY (2005). Arabidopsis calcium-dependent protein kinase AtCPK32 interacts with ABF4, a transcriptional regulator of abscisic acid-responsive gene expression, and modulates its activity. Plant Physiol.

[17] Kim SY, Nam KH (2010). Physiological roles of ERD10 in abiotic stresses and seed germination of Arabidopsis. Plant Cell Reports.

[18] Kovacs D, Kalmar E, Torok Z, Tompa P (2008). Chaperone activity of ERD10 and ERD14, two disordered stress-related plant proteins. Plant Physiol.

[19] Abe H, Yamaguchi-Shinozaki K, Urao T, Iwasaki T, Hosokawa D, Shinozaki K (1997). Role of arabidopsis MYC and MYB homologs in drought- and abscisic acid-regulated gene expression. Plant Cell.

[20] Ryu HY, Kim SY, Park HM, You JY, Kim BH, Lee JS, Nam KH (2009). Modulations of AtGSTF10 expression induce stress tolerance and BAK1-mediated cell death. Biochem Biophys Res Commun.

[21] Duque P (2011). A role for SR proteins in plant stress responses. Plant Signal Behav.

[22] Barta A, Kalyna M, Reddy AS (2010). Implementing a rational and consistent nomenclature for serine/arginine-rich protein splicing factors (SR proteins) in plants. Plant Cell.

[23] Reddy AS (2007). Alternative splicing of pre-messenger RNAs in plants in the genomic era. Annu Rev Plant Biol.

[24] Reddy AS, Shad Ali G (2011). Plant serine/arginine-rich proteins: roles in precursor messenger RNA splicing, plant development, and stress responses. Wiley Interdiscip Rev RNA.

[25] Li W, Lin WD, Ray P, Lan P, Schmidt W (2013). Genome-wide detection of condition-sensitive alternative splicing in Arabidopsis roots. Plant Physiol.

[26] Cui P, Zhang S, Ding F, Ali S, Xiong L (2014). Dynamic regulation of genome-wide pre-mRNA splicing and stress tolerance by the Sm-like protein LSm5 in Arabidopsis. Genome Biol.

[27] Forne T, Labourier E, Antoine E, Rossi F, Gallouzi I, Cathala G, Tazi J, Brunel C (1996). Structural features of U6 snRNA and dynamic interactions with other spliceosomal components leading to pre-mRNA splicing. Biochimie.

[28] Braunschweig U, Gueroussov S, Plocik AM, Graveley BR, Blencowe BJ (2013). Dynamic integration of splicing within gene regulatory pathways. Cell.

[29] Lesser CF, Guthrie C (1993). Mutations in U6 snRNA that alter splice-site specificity - implications for the active-site. Science.

[30] Xiong L, Schumaker KS, Zhu JK (2002). Cell signaling during cold, drought, and salt stress. Plant Cell.

[31] Yamaguchi-Shinozaki K, Shinozaki K (2006). Transcriptional regulatory networks in cellular responses and tolerance to dehydration and cold stresses. Annu Rev Plant Biol.

[32] Isshiki M, Tsumoto A, Shimamoto K (2006). The serine/arginine-rich protein family in rice plays important roles in constitutive and alternative splicing of pre-mRNA. Plant Cell.

[33] Palusa SG, Ali GS, Reddy ASN (2007). Alternative splicing of pre-mRNAs of Arabidopsis serine/arginine-rich proteins: regulation by hormones and stresses. Plant Journal.

[34] Chen T, Cui P, Chen H, Ali S, Zhang S, Xiong L (2013). A KH-domain RNA-binding protein interacts with FIERY2/CTD phosphatase-like 1 and splicing factors and is important for pre-mRNA splicing in Arabidopsis. PLoS Genetics.

[35] Xiong L, Lee B, Ishitani M, Lee H, Zhang C, Zhu JK (2001). *FIERY1* encoding an inositol polyphosphate 1-phosphatase is a negative regulator of abscisic acid and stress signaling in Arabidopsis. Genes Dev.

[36] Trapnell C, Pachter L, Salzberg SL (2009). TopHat: discovering splice junctions with RNA-Seq. Bioinformatics.

[37] Trapnell C, Williams BA, Pertea G, Mortazavi A, Kwan G, van Baren MJ, Salzberg SL, Wold BJ, Pachter L (2010). Transcript assembly and quantification by RNA-Seq reveals unannotated transcripts and isoform switching during cell differentiation. Nat Biotechnol.

[38] Brooks AN, Yang L, Duff MO, Hansen KD, Park JW, Dudoit S, Brenner SE, Graveley BR (2011). Conservation of an RNA regulatory map between *Drosophila* and mammals. Genome Res.

