# Supplementary figures and images for: Genome-wide analysis of alternative splicing of pre-mRNA under salt stress in Arabidopsis
Source: BMC Genomics. 2014 Jun 4;15(1):431. doi: 10.1186/1471-2164-15-431 (PMC4079960; doi:10.1186/1471-2164-15-431)

### Control

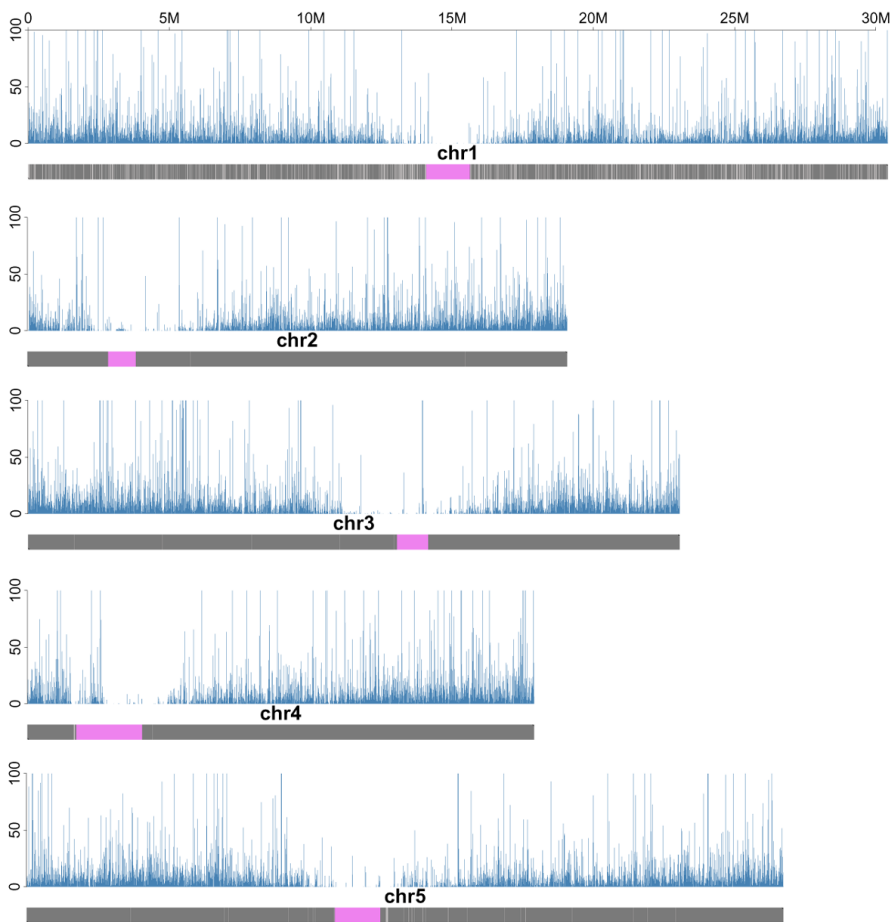

### 50 mM NaCl

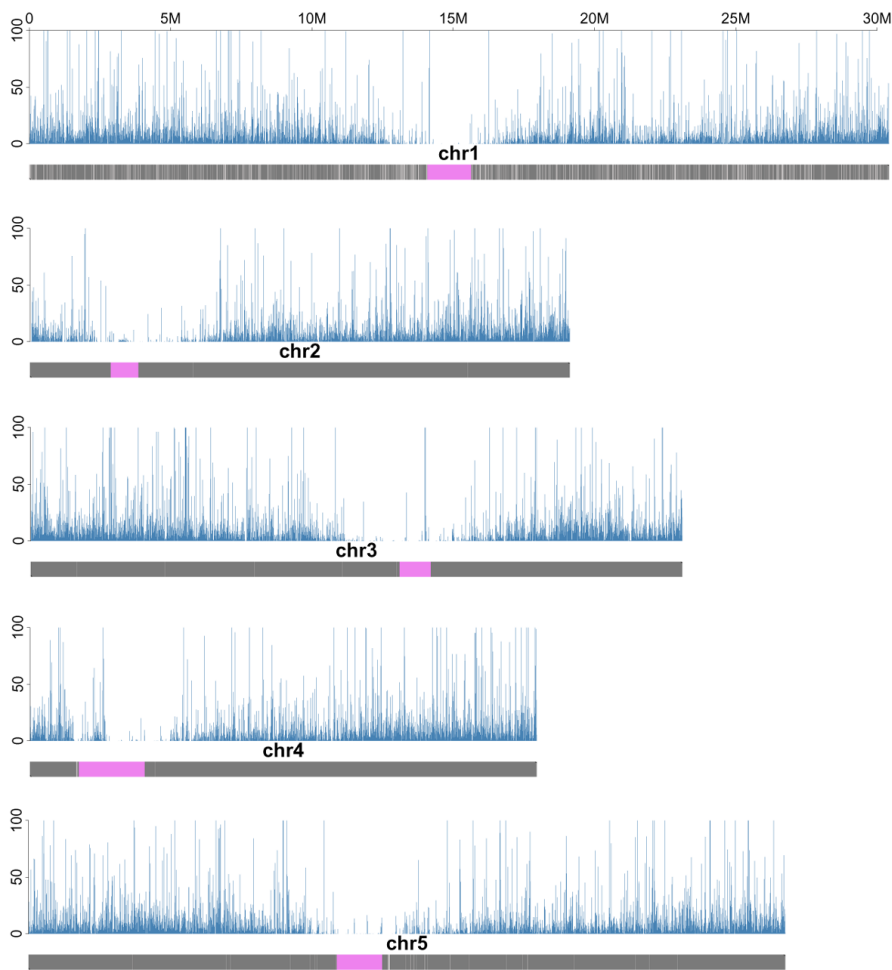

### 150 mM NaCl

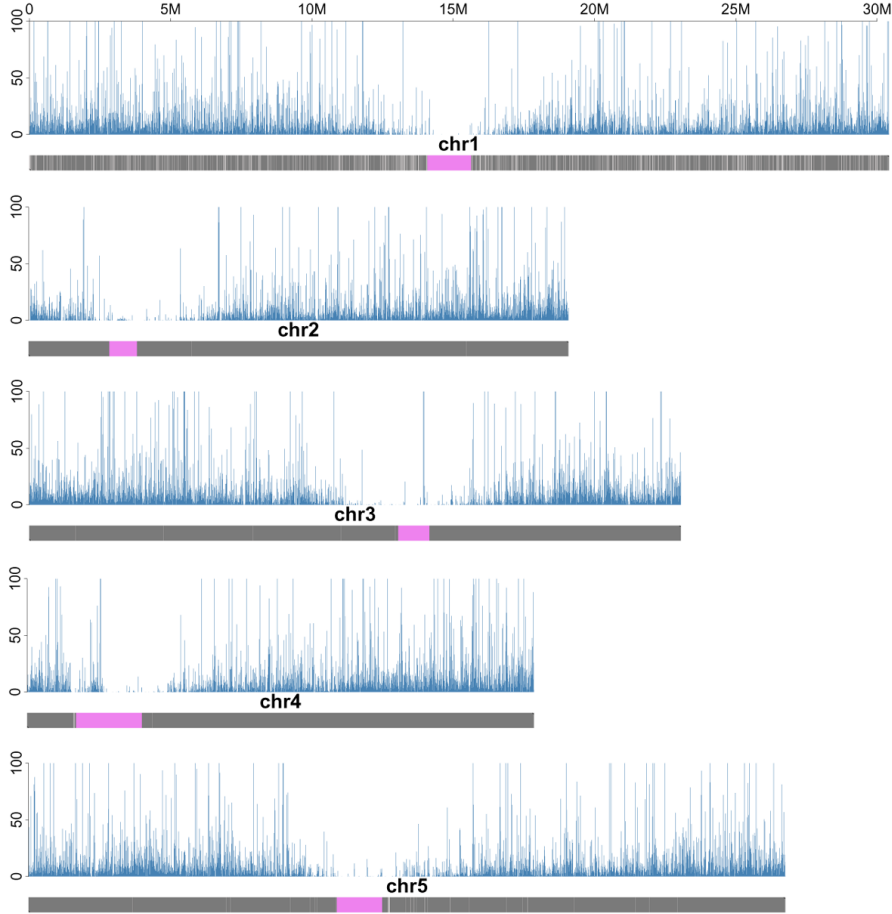

Supplement: Supplementary file 2 — Additional file 2: Transcription profiles in the control, 50 or 150 NaCl treatments were plotted across the Arabidopsis genome. Distribution of the RNA-seq read density along the chromosome length is shown. Each vertical blue bar represents log2 of the frequency of reads plotted against the chromosome coordinates. A schematic drawing of the chromosome and its features is shown below the read density. Approximate boundaries of Arabidopsis centromeres are depicted in violet. (PDF 954 KB) [file 12864_2014_6180_MOESM2_ESM.pdf]

alternative 5'SSs

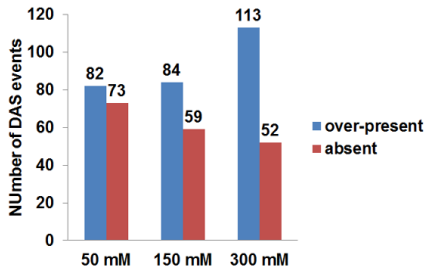

alternative 3'SSs

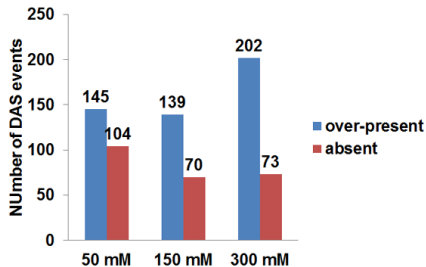

exon skipping

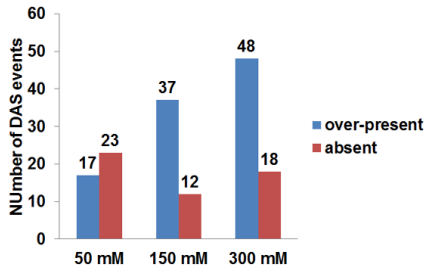

intron retention

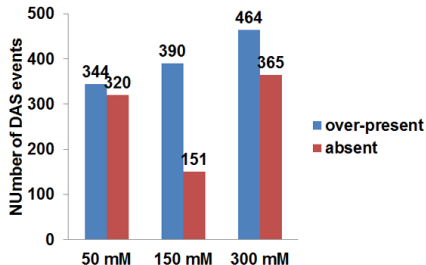

Supplement: Supplementary file 4 — Additional file 4: The number of DAS events in 50, 150 or 300 mM NaCl treatments. Blue bars indicate the number of DAS events that are significantly over-represented in NaCl treatment plants. Red bars indicate the number of DAS events that are absent in NaCl treatment plants. (PDF 179 KB) [file 12864_2014_6180_MOESM4_ESM.pdf]

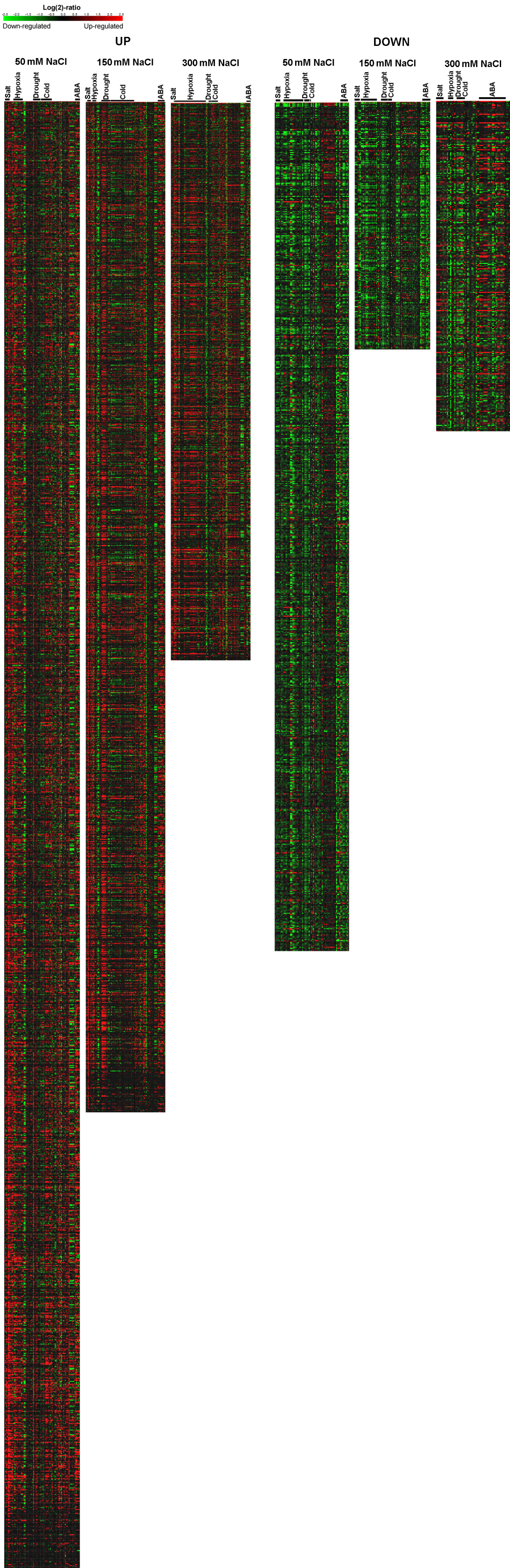

Supplement: Supplementary file 18 — Additional file 18: The heatmaps generated by mapping the DE genes in the 50, 150 or 300 mM NaCl treatments to the database of microarray data with Genevestigator. The heatmaps indicate that DE genes (including the up-regulated and down-regulated genes) in NaCl treatments are consistent with the gene profiles derived from the microarray data. (PDF 8 MB) [file 12864_2014_6180_MOESM18_ESM.pdf]

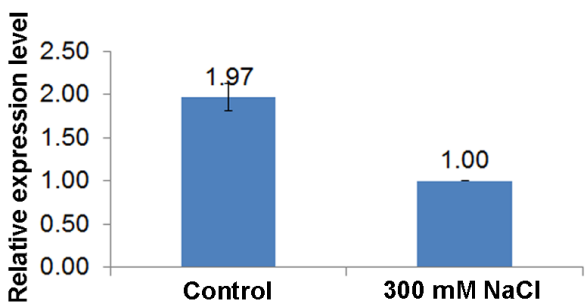

Supplement: Supplementary file 22 — Additional file 22: U6 snRNA expression levels as determined by quantitative RT-PCR. The snRNA level in NaCl treatment plants was lower than in the control plants. (PDF 50 KB) [file 12864_2014_6180_MOESM22_ESM.pdf]

**A**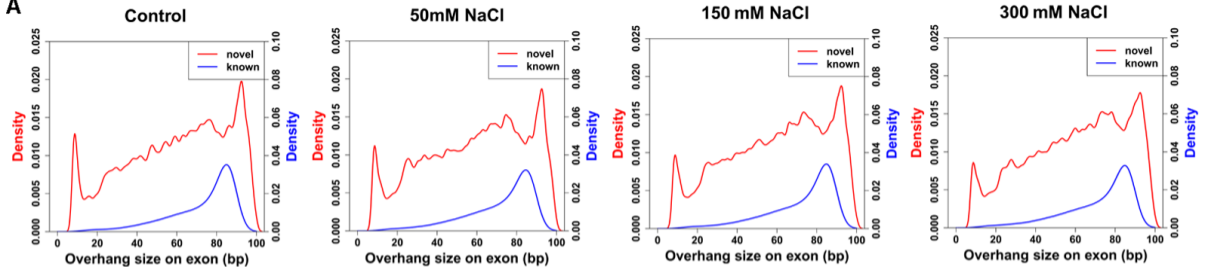**B**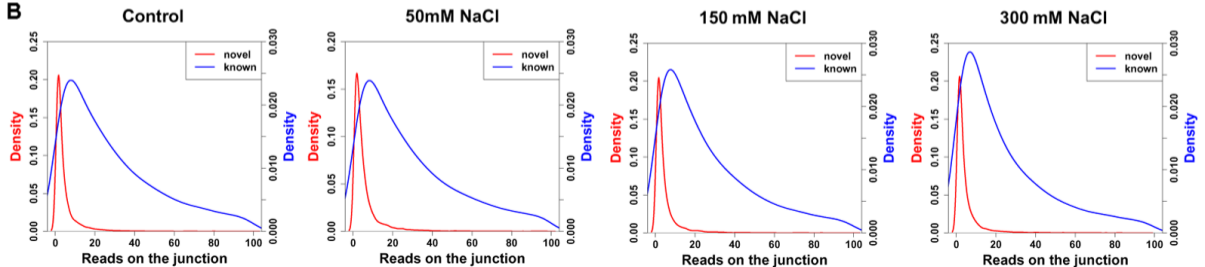

Supplement: Supplementary file 23 — Additional file 23: The distinctive features between known and novel splice junctions. (A) The density of the overhang size with exons for known and novel splice junctions in each sample. The x-axis indicates the size of the overhang with exon and the y-axis indicates the density of the sizes. A great number of novel junctions has shorter overhangs (i.e., fewer than 20 bp) with the corresponding exons, while most of the annotated junctions have larger overhang size, with the enrichment at ~90 bp. (B) The density of junction-read coverage for known and novel junctions. The novel junctions have relatively low coverage compared to the annotated junctions. (PDF 382 KB) [file 12864_2014_6180_MOESM23_ESM.pdf]

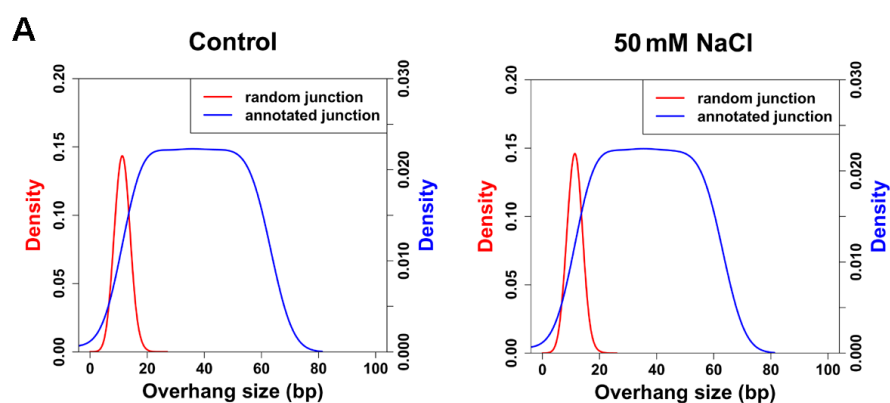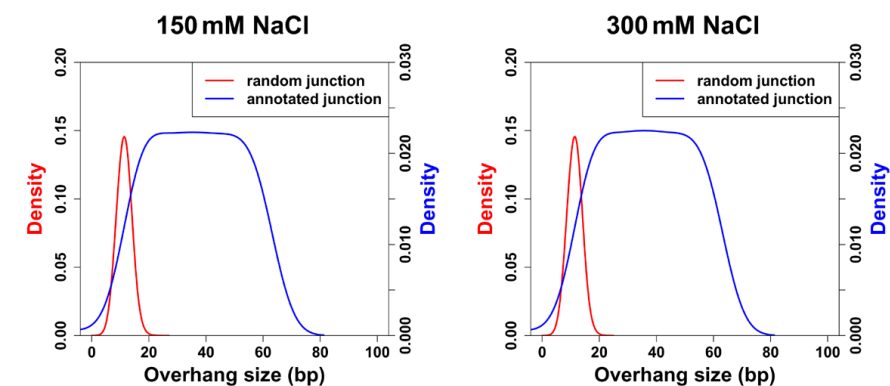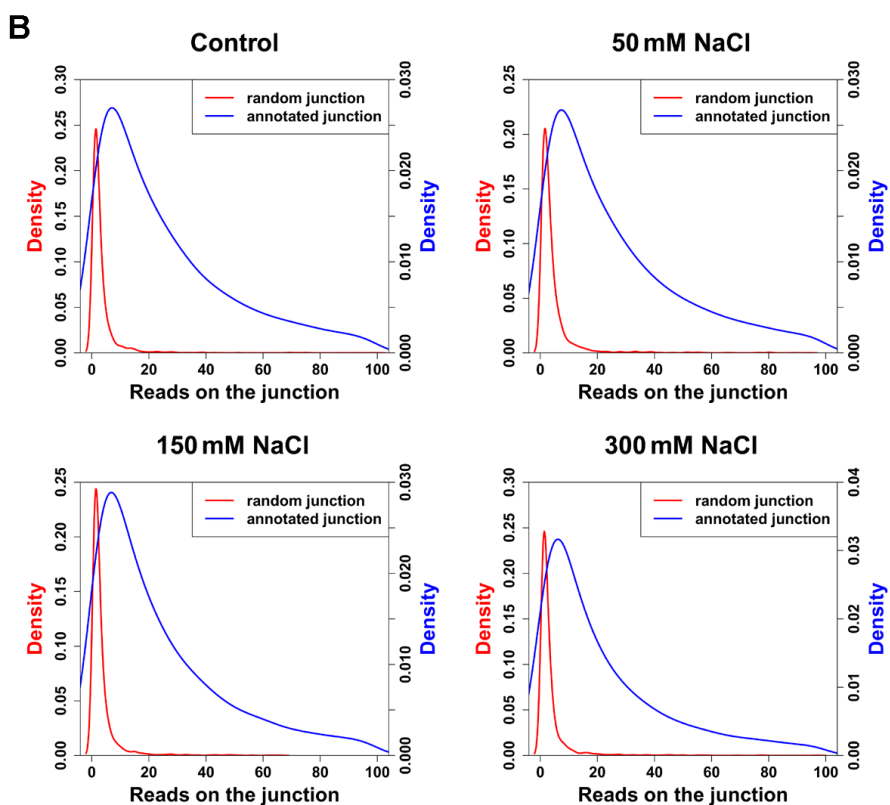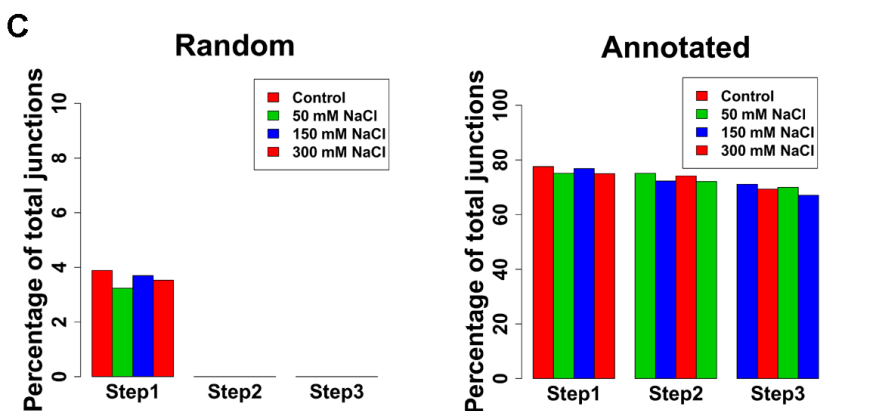

Supplement: Supplementary file 24 — Additional file 24: The features of false positive (random) and annotated junctions. (A) The density of the overhang size of false positive and annotated junctions. Most of false positive junctions have shorter overhang sizes, while the annotated junctions have larger overhang sizes. (B) The density of junction-read coverage of false positives and annotated junctions. More than half of false positive junctions have only one read spanning the junction, while the annotated junctions have higher reads coverage. (C) Distinguishing true junctions from false positive alignments. To reduce the number of false positive junctions, as determined by randomly generated junctions, we required that the overhang size must be more than 20 bp with at least two reads spanning the junctions. Using both criteria, the false positive junctions sharply reduced to very low levels (close to zero). In contrast, the annotated junctions show no obvious decrease. (PDF 552 KB) [file 12864_2014_6180_MOESM24_ESM.pdf]
