# Supplementary material for: Genome-wide analysis of alternative splicing of pre-mRNA under salt stress in Arabidopsis
Source: BMC Genomics. 2014 Jun 4;15(1):431. doi: 10.1186/1471-2164-15-431 (PMC4079960; doi:10.1186/1471-2164-15-431)
Supplement: Supplementary file 14 — Additional file 14: A two-dimensional (2-D) view of the relationship between the genes with abnormal splicing and their functional annotations in 50, 150 or 300 mM NaCl treatments. The functional classification of genes was done by the DAVID software. The top 20 functional annotations that were ordered by the enrichment scores were selected for the 2-D view, which indicates that genes with abnormal splicing were strikingly enriched in the response-to-abiotic-stress category. (PDF 2 MB) [file 12864_2014_6180_MOESM14_ESM.pdf]

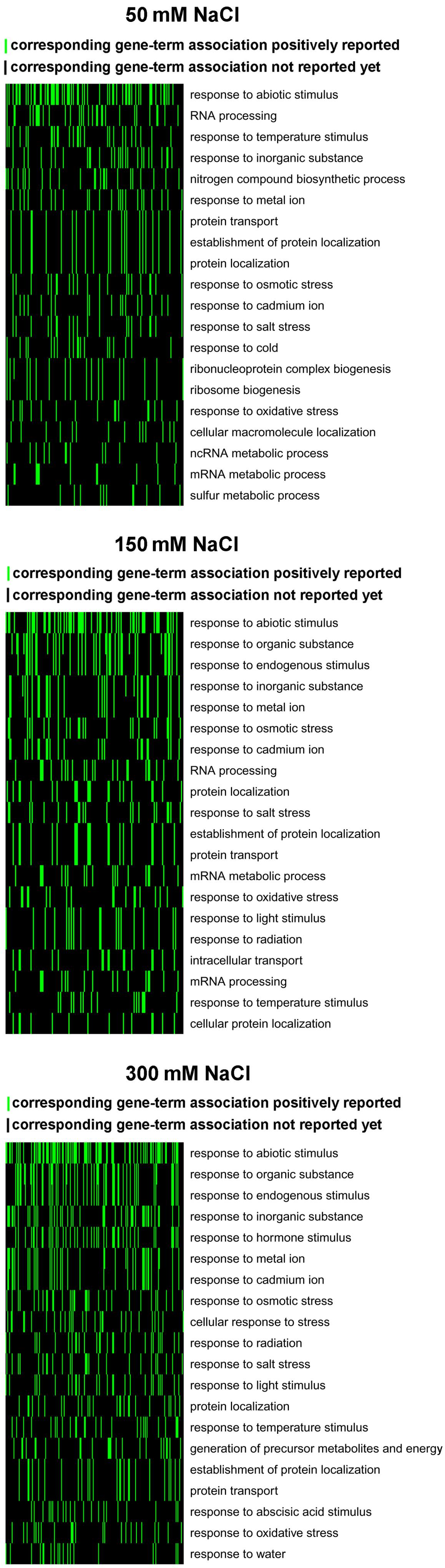
50 mM NaCl
corresponding gene-term association positively reported
corresponding gene-term association not reported yet
response to abiotic stimulus
RNA processing
response to temperature stimulus
response to inorganic substance
nitrogen compound biosynthetic process
response to metal ion
protein transport
establishment of protein localization
protein localization
response to osmotic stress
response to cadmium ion
response to salt stress
response to cold
ribonucleoprotein complex biogenesis
ribosome biogenesis
response to oxidative stress
cellular macromolecule localization
ncRNA metabolic process
mRNA metabolic process
sulfur metabolic process
150 mM NaCl
corresponding gene-term association positively reported
corresponding gene-term association not reported yet
response to abiotic stimulus
response to organic substance
response to endogenous stimulus
response to inorganic substance
response to metal ion
response to osmotic stress
response to cadmium ion
RNA processing
protein localization
response to salt stress
establishment of protein localization
protein transport
mRNA metabolic process
response to oxidative stress
response to light stimulus
response to radiation
intracellular transport
mRNA processing
response to temperature stimulus
cellular protein localization
300 mM NaCl
corresponding gene-term association positively reported
corresponding gene-term association not reported yet
response to abiotic stimulus
response to organic substance
response to endogenous stimulus
response to inorganic substance
response to hormone stimulus
response to metal ion
response to cadmium ion
response to osmotic stress
cellular response to stress
response to radiation
response to salt stress
response to light stimulus
protein localization
response to temperature stimulus
generation of precursor metabolites and energy
establishment of protein localization
protein transport
response to abscisic acid stimulus
response to oxidative stress
response to water
