# Supplementary material for: Genome-wide analysis of alternative splicing of pre-mRNA under salt stress in Arabidopsis
Source: BMC Genomics. 2014 Jun 4;15(1):431. doi: 10.1186/1471-2164-15-431 (PMC4079960; doi:10.1186/1471-2164-15-431)
Supplement: Supplementary file 15 — Additional file 15: A network generated by Mapman indicates that genes with aberrant splicing in the 50, 150 or 300 mM NaCl treatments were involved in various stress response pathways, including hormone-signaling pathways, MAPK-signaling pathways and transcription regulation. (PDF 740 KB) [file 12864_2014_6180_MOESM15_ESM.pdf]

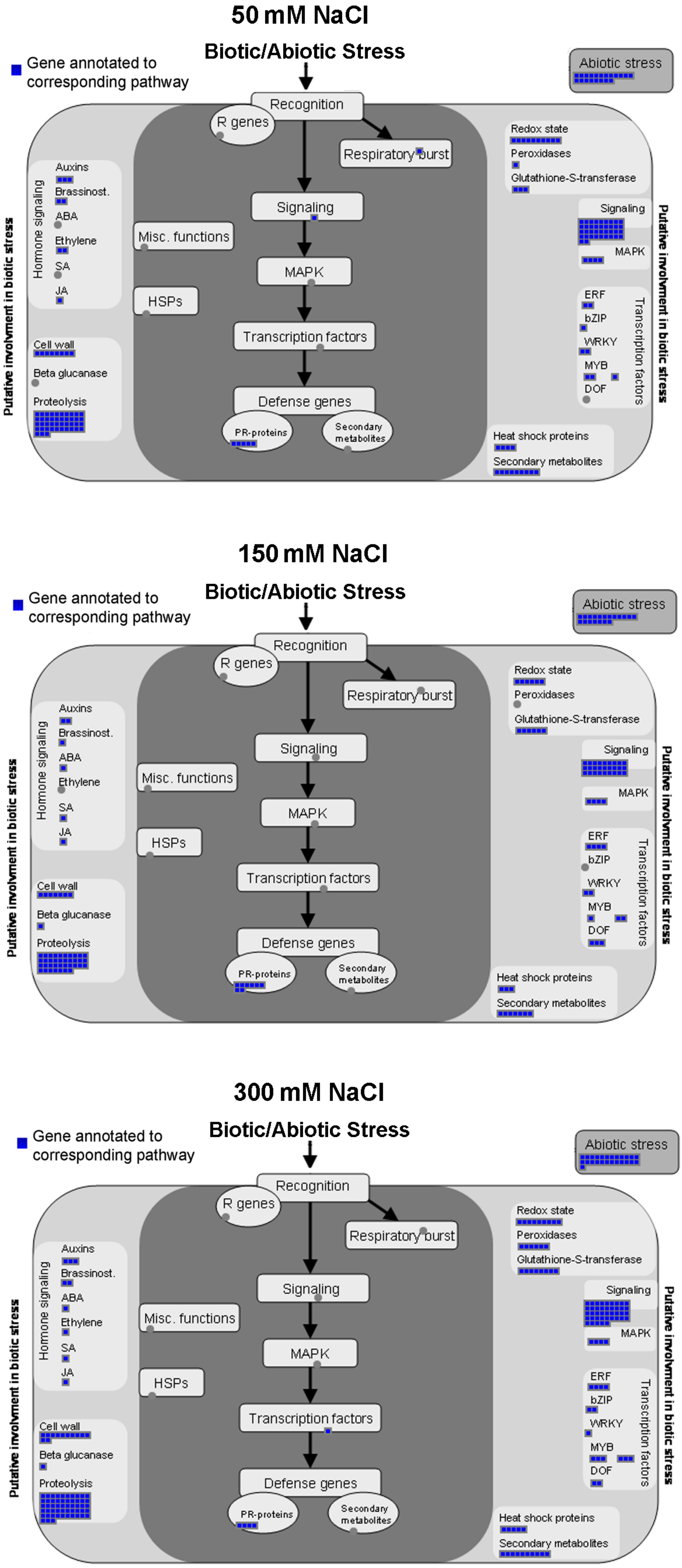
50 mM NaCl
Biotic/Abiotic Stress
Gene annotated to corresponding pathway
Abiotic stress
Recognition
R genes
Respiratory burst
Signaling
Misc. functions
MAPK
HSPs
Transcription factors
Defense genes
PR-proteins
Secondary metabolites
Putative involvment in biotic stress
Hormone signaling
Auxins
Brassinost.
ABA
Ethylene
SA
JA
Cell wall
Beta glucanase
Proteolysis
Redox state
Peroxidases
Glutathione-S-transferase
Signaling
MAPK
ERF
bZIP
WRKY
MYB
DOF
Transcription factors
Heat shock proteins
Secondary metabolites
Putative involvment in biotic stress
150 mM NaCl
Biotic/Abiotic Stress
Gene annotated to corresponding pathway
Abiotic stress
Recognition
R genes
Respiratory burst
Signaling
Misc. functions
MAPK
HSPs
Transcription factors
Defense genes
PR-proteins
Secondary metabolites
Putative involvment in biotic stress
Hormone signaling
Auxins
Brassinost.
ABA
Ethylene
SA
JA
Cell wall
Beta glucanase
Proteolysis
Redox state
Peroxidases
Glutathione-S-transferase
Signaling
MAPK
ERF
bZIP
WRKY
MYB
DOF
Transcription factors
Heat shock proteins
Secondary metabolites
Putative involvment in biotic stress
300 mM NaCl
Biotic/Abiotic Stress
Gene annotated to corresponding pathway
Abiotic stress
Recognition
R genes
Respiratory burst
Signaling
Misc. functions
MAPK
HSPs
Transcription factors
Defense genes
PR-proteins
Secondary metabolites
Putative involvment in biotic stress
Hormone signaling
Auxins
Brassinost.
ABA
Ethylene
SA
JA
Cell wall
Beta glucanase
Proteolysis
Redox state
Peroxidases
Glutathione-S-transferase
Signaling
MAPK
ERF
bZIP
WRKY
MYB
DOF
Transcription factors
Heat shock proteins
Secondary metabolites
Putative involvment in biotic stress
